# Kounis Syndrome Without Systemic Allergy: A Subtle Presentation of Stent Thrombosis

**DOI:** 10.7759/cureus.84638

**Published:** 2025-05-22

**Authors:** Giovanni Paolella, Viktoriya Bikeyeva, Mae Leef, George C Michalopoulos, Joseph Adams, Andrii Labchuk, Adib Chaus

**Affiliations:** 1 Internal Medicine, Advocate Lutheran General Hospital, Park Ridge, USA; 2 Cardiology, Advocate Lutheran General Hospital, Park Ridge, USA

**Keywords:** allergic acute coronary syndrome, contrast allergy, kounis syndrome, st-elevation myocardial infarction, stent thrombosis, type iii kounis

## Abstract

Kounis Syndrome, or allergic acute coronary syndrome, is a rare cause of myocardial ischemia triggered by hypersensitivity reactions. Type III Kounis Syndrome, involving in-stent thrombosis following an allergic insult, is particularly rare and underrecognized. We report the case of a 67-year-old woman with a known contrast allergy who developed inferior ST-elevation myocardial infarction (STEMI) complicated by early stent thrombosis, suspected to be due to type III Kounis Syndrome. This case underscores the importance of maintaining a high index of suspicion for allergic triggers in acute coronary syndromes and considering contrast-sparing strategies in high-risk individuals.

## Introduction

Kounis Syndrome describes acute coronary events triggered by hypersensitivity reactions. First reported in 1991, it is classified into three types: Type I (coronary vasospasm without underlying coronary artery disease), type II (plaque rupture in diseased arteries), and type III (in-stent thrombosis secondary to hypersensitivity) [1]. Hypersensitivity reactions are categorized into four types, with Kounis Syndrome typically resulting from type I (IgE-mediated, immediate hypersensitivity) or type III (immune complex-mediated) reactions [2]. Type III Kounis Syndrome is particularly rare, requiring the coexistence of coronary stent placement and a significant allergic response capable of provoking stent thrombosis.

Although precise incidence rates are difficult to establish, Kounis Syndrome is estimated to account for approximately 1.1% to 3.4% of all acute coronary syndrome presentations in some studies [2]. However, type III Kounis Syndrome, involving stent thrombosis, is exceptionally rare, with only scattered case reports and small case series described in the literature. It remains likely underdiagnosed due to its often-subtle clinical presentation and overlap with more common procedural complications.

Given the increasing prevalence of coronary interventions and drug-eluting stent usage, awareness of Kounis Syndrome, particularly the rare type III variant, is critical for timely diagnosis and management [3].

## Case presentation

A 67-year-old female with a history of type 2 diabetes, hyperlipidemia, hypothyroidism, and a documented anaphylactic reaction to iodinated contrast presented with substernal chest pressure, rated 5-7/10, radiating to her left shoulder. The pain began at rest, worsened with exertion, and was associated with nausea but no diaphoresis or shortness of breath.

**Figure 1:**
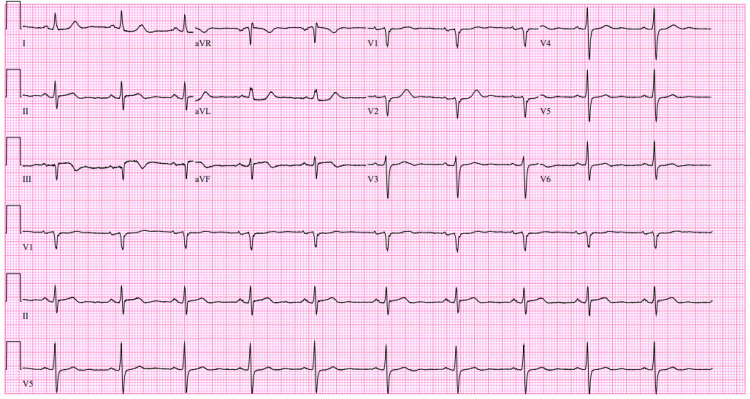
Initial 12-lead ECG demonstrating ST-segment elevations in the inferior leads, consistent with inferior STEMI. STEMI: ST-elevation myocardial infarction

An initial ECG revealed ST-elevation in the inferior leads (Figure 1).

**Figure 2:**
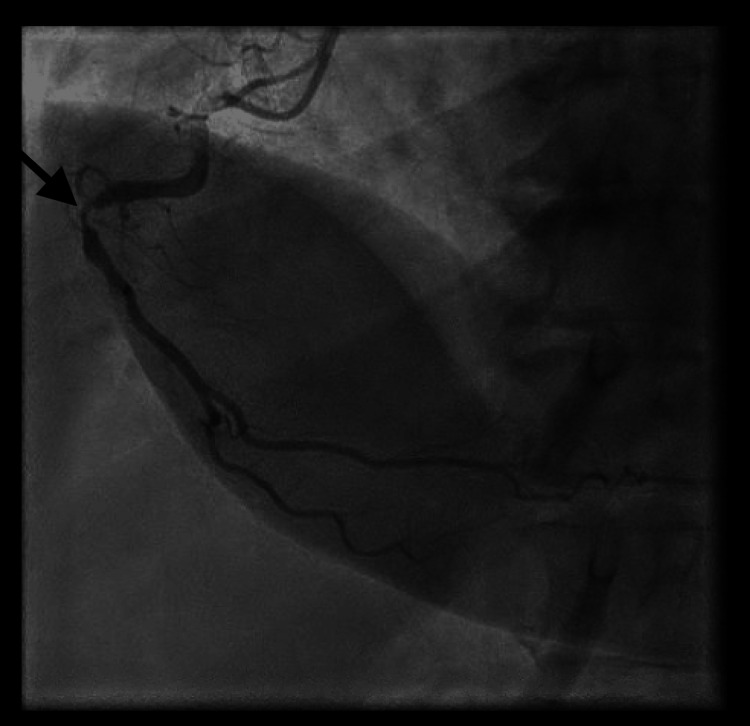
Diagnostic coronary angiogram showing 99% proximal stenosis of the right coronary artery.

Troponin peaked at 1.7 ng/mL. Due to her known contrast allergy, she was premedicated with 200 mg intravenous methylprednisolone and 50 mg diphenhydramine prior to emergent coronary angiography. Diagnostic angiography showed a critical 99% proximal right coronary artery (RCA) stenosis (Figure 2).

**Figure 3:**
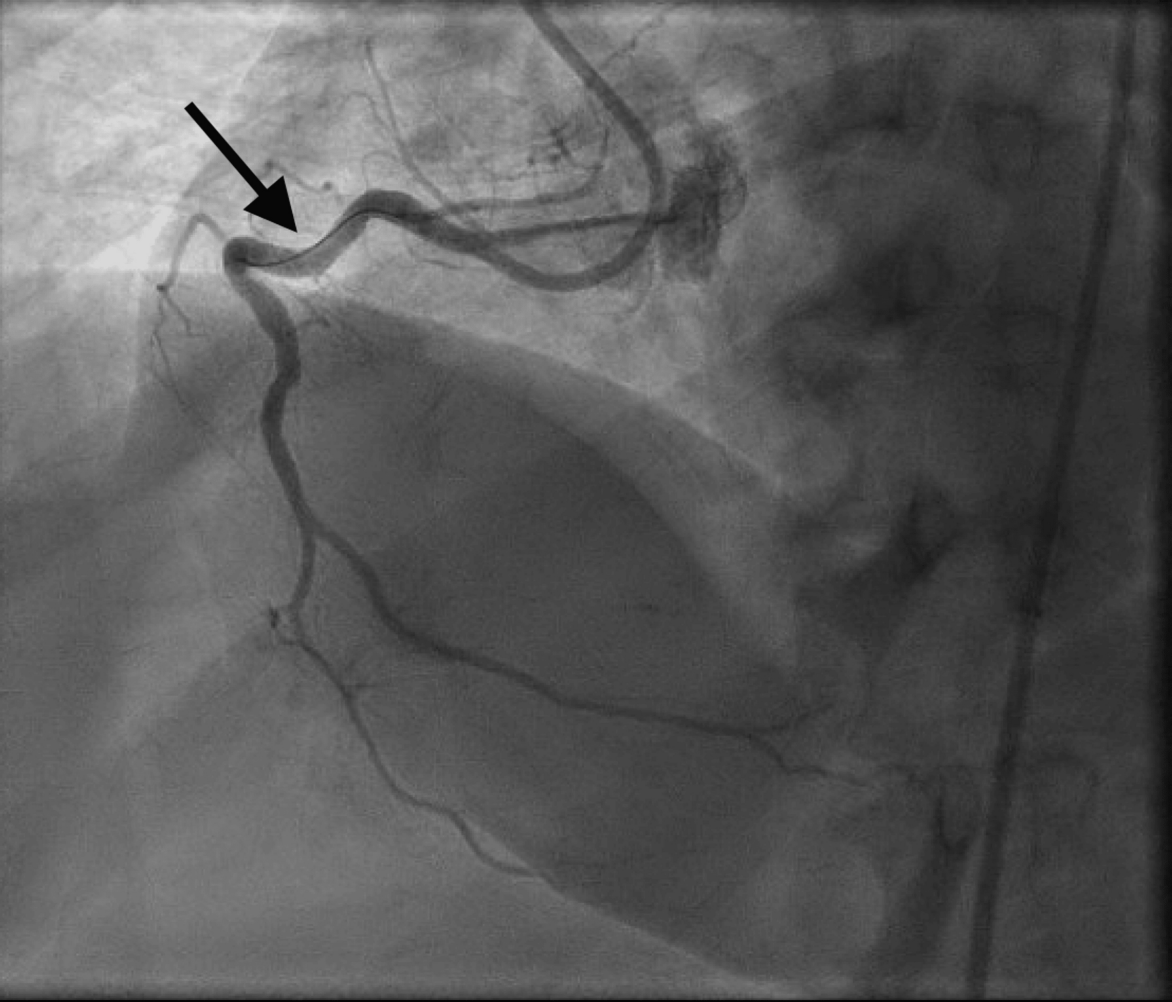
Post-intervention angiogram demonstrating successful stent placement in the RCA with TIMI-3 flow and no residual stenosis. RCA: Right coronary artery

A 3.0 × 30 mm Orsiro drug-eluting stent (DES) was placed following pre-dilation and post-dilation, achieving thrombolysis in myocardial infarction (TIMI-3) flow (Figure 3).

She was loaded with prasugrel 60 mg and continued dual antiplatelet therapy (DAPT) with aspirin and prasugrel.

**Figure 4:**
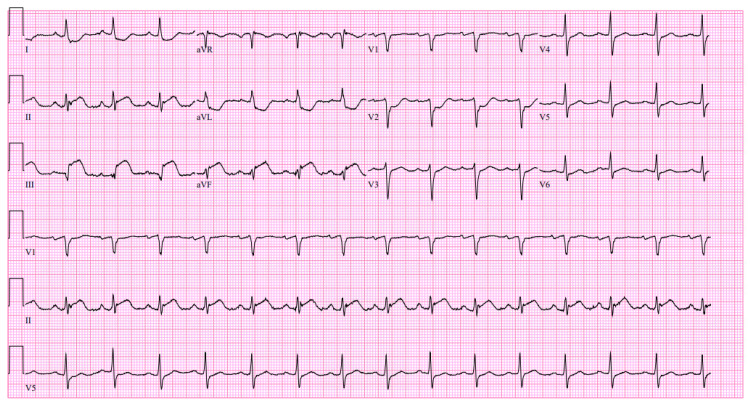
Repeat ECG following symptom recurrence showing persistent inferior ST-segment elevations.

One hour after the procedure, the patient developed acute worsening chest pain with persistent 7/10 intensity, associated with dizziness and hypotension. Repeat ECG revealed worsening inferior ST-segment elevations (Figure 4).

**Figure 5:**
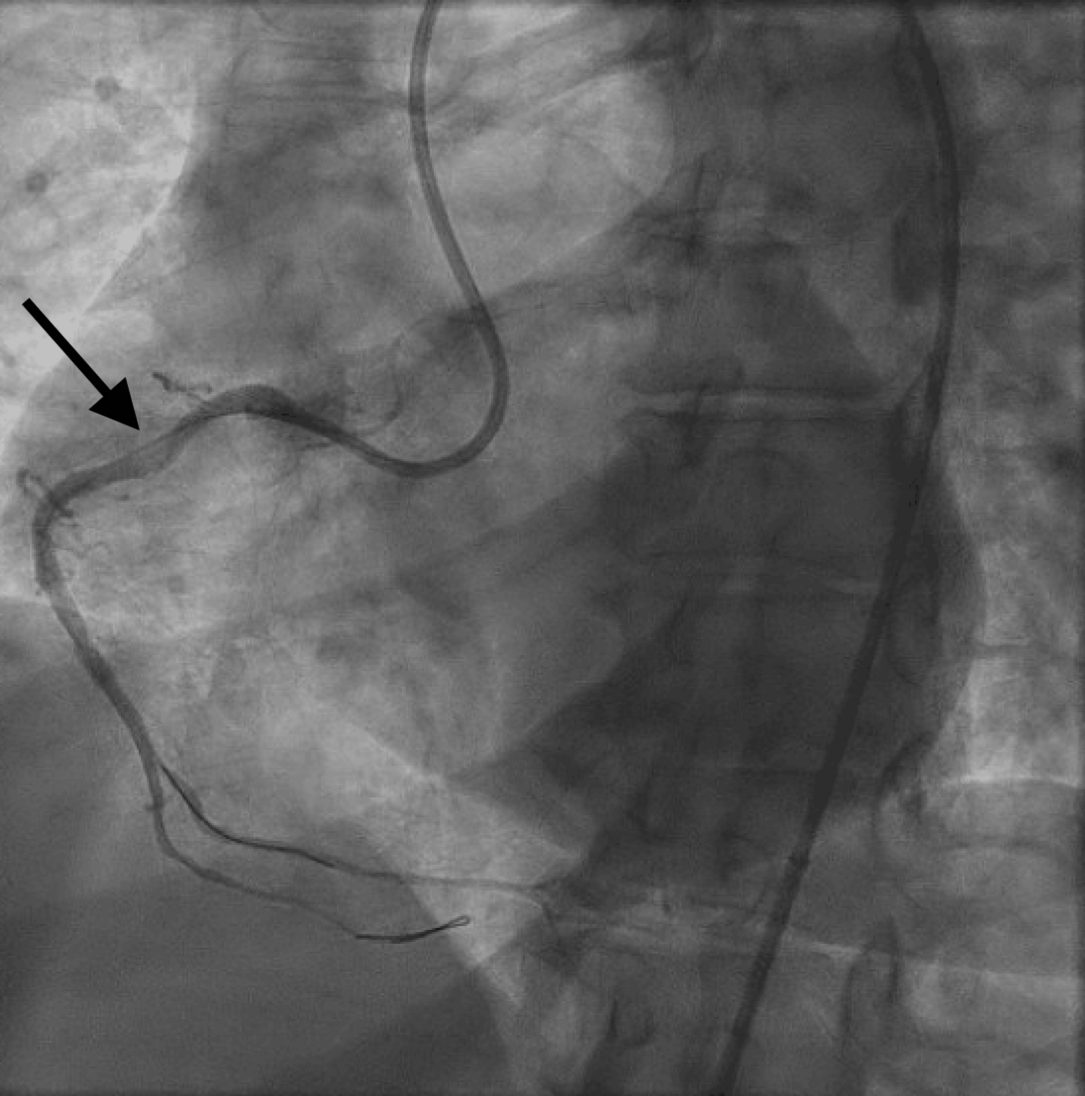
Coronary angiogram revealing acute in-stent thrombosis in the proximal RCA. RCA: Right coronary artery

Emergent repeat angiography revealed acute proximal RCA stent thrombosis (Figure 5).

**Figure 6:**
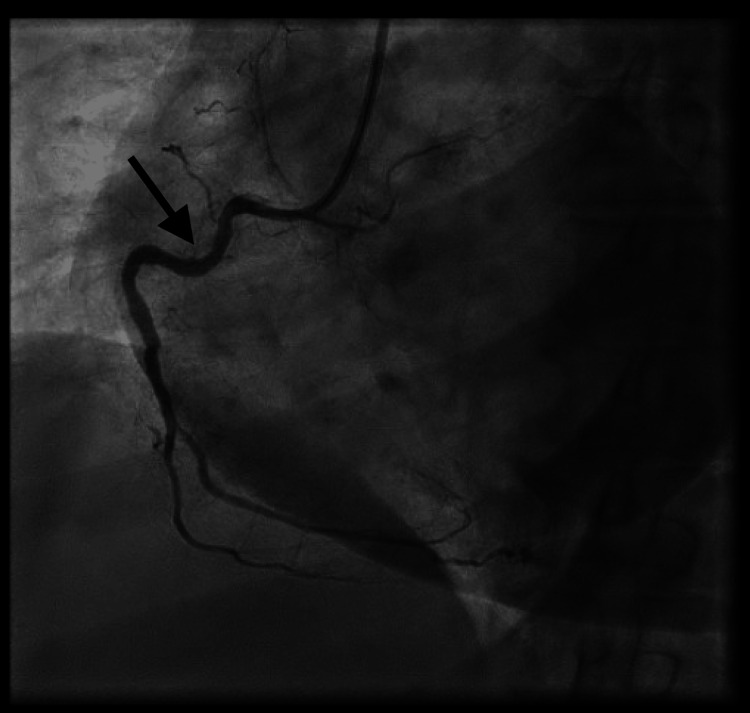
Final angiographic result after placement of a second overlapping drug-eluting stent showing restored TIMI-3 flow. TIMI: Thrombolysis in myocardial infarction

After difficult lesion crossing, serial balloon angioplasty was performed, followed by placement of a second overlapping 3.0 × 26 mm Orsiro DES. Optical coherence tomography (OCT) demonstrated well-apposed stents without evidence of mechanical issues such as malapposition, underexpansion, or edge dissection. A minor distal wire perforation was noted but was hemodynamically insignificant (Figure 6). 

Post-procedure, the patient developed a retroperitoneal hematoma requiring two units of blood transfusion. Anticoagulation with heparin had been discontinued per standard post-PCI protocol following DES placement. Eptifibatide was administered for a planned 12-hour duration and completed as scheduled. Echocardiography showed a preserved ejection fraction of 59% with regional wall motion abnormalities. Further PCI to the left anterior descending artery (LAD) was deferred, given her high thrombotic and bleeding risk profile. She was discharged on medical therapy with outpatient cardiology follow-up.

## Discussion

This case exemplifies type III Kounis Syndrome, where early in-stent thrombosis is likely provoked by an allergic response to iodinated contrast. While systemic allergic manifestations were absent, the strong temporal relationship between contrast exposure and the thrombotic event, alongside exclusion of mechanical or technical PCI failures, supports the diagnosis. Notably, patients with Kounis Syndrome often do not exhibit classical allergic symptoms, complicating recognition and delaying treatment [4].

Although premedication protocols using corticosteroids and antihistamines are routinely employed in patients with contrast allergy, their efficacy in fully preventing Kounis-related complications remains unclear [5]. Despite receiving standard premedication, our patient developed a serious thrombotic event, underscoring the limitations of prophylactic measures alone.

Alternative differential diagnoses, including mechanical stent complications (such as malapposition or underexpansion), spontaneous coronary artery dissection (SCAD), hypercoagulable states, heparin-induced thrombocytopenia (HIT), and progression of native atherosclerotic disease, were considered. However, OCT imaging demonstrated well-apposed stents without evidence of mechanical failure, and the patient’s laboratory values, including platelet count and coagulation studies, were unremarkable. The focused localization of ischemia to the treated vessel and absence of thrombocytopenia or systemic hypercoagulability made other causes less likely.

Type III Kounis Syndrome remains rare, owing to the requirement for both a coronary stent and a significant hypersensitivity reaction capable of triggering in-situ thrombosis. Most reported cases involve DES, suggesting that the stent surface itself or hypersensitivity to contrast material may serve as the antigenic stimulus. This syndrome is often underdiagnosed, and its contribution to major adverse cardiac events (MACE) is likely underappreciated, given the lack of large-scale outcome data.

Management of Kounis Syndrome poses significant challenges. In the setting of emergent coronary intervention, avoidance of iodinated contrast is rarely feasible. Strategies such as careful premedication, minimizing contrast volume, considering iso-osmolar contrast agents, and the use of adjunctive imaging techniques (e.g., intravascular ultrasound or OCT without contrast flushes) in select cases may mitigate risk. Epinephrine remains reserved for the acute treatment of anaphylaxis and is not recommended for prophylactic use during PCI.

Early recognition of Kounis Syndrome during or after coronary interventions is essential to guiding immediate management, preventing recurrence, and modifying future procedural plans. In patients with known severe contrast allergies undergoing PCI, heightened vigilance is warranted, even when standard prophylactic measures have been applied.

## Conclusions

This case underscores the importance of recognizing type III Kounis Syndrome in patients undergoing coronary interventions. Despite appropriate premedication, hypersensitivity-mediated stent thrombosis can occur without overt systemic signs. Broader learning points include the necessity for vigilance even when prophylactic measures are taken, considering alternative imaging strategies in patients with significant contrast allergies, and understanding that allergic triggers can lead to severe procedural complications. Early recognition and appropriate management are essential to improve outcomes and prevent further cardiac events.
